# Prognostic implications of CD9 in childhood acute lymphoblastic leukemia: insights from a nationwide multicenter study in China

**DOI:** 10.1038/s41375-023-02089-3

**Published:** 2023-11-25

**Authors:** Kam Tong Leung, Jiaoyang Cai, Yu Liu, Kathy Yuen Yee Chan, Jingbo Shao, Hui Yang, Qun Hu, Yao Xue, Xuedong Wu, Xia Guo, Xiaowen Zhai, Ningling Wang, Xue Li, Xin Tian, Zheng Li, Ning Xue, Yuxia Guo, Lingzhen Wang, Yao Zou, Peifang Xiao, Yingyi He, Runming Jin, Jingyan Tang, Jun J. Yang, Shuhong Shen, Ching-Hon Pui, Chi Kong Li

**Affiliations:** 1 Department of Paediatrics, The Chinese University of Hong Kong, Shatin, Hong Kong; 2 Hong Kong Hub of Paediatric Excellence, The Chinese University of Hong Kong, Shatin, Hong Kong; 3 Department of Hematology/Oncology, Shanghai Children’s Medical Center, School of Medicine, Shanghai Jiao Tong University, National Health Committee Key Laboratory of Pediatric Hematology & Oncology, Shanghai, China; 4 Pediatric Translational Medicine Institute, Shanghai Children’s Medical Center, School of Medicine, Shanghai Jiao Tong University, National Health Committee Key Laboratory of Pediatric Hematology & Oncology, Shanghai, China; 5 Department of Hematology/Oncology, Shanghai Children’s Hospital, Shanghai, China; 6 Department of Pediatrics, Xiangya Hospital Central South University, Changsha, China; 7 Department of Pediatrics, Tongji Hospital of Tongji Medical College, Huazhong University of Science and Technology, Wuhan, China; 8 Department of Hematology/Oncology, Children’s Hospital of Nanjing Medical University, Nanjing, China; 9 Department of Pediatrics, Nanfang Hospital, Southern Medical University, Guangzhou, China; 10 Department of Pediatrics, West China Second University Hospital, Sichuan University, Key Laboratory of Birth Defects and Related Disease of Women and Children, Ministry of Education, Chengdu, China; 11 Department of Hematology/Oncology, Children’s Hospital of Fudan University, Shanghai, China; 12 Department of Pediatrics, Anhui Medical University Second Affiliated Hospital, Anhui, China; 13 Department of Pediatrics, Qilu Hospital of Shandong University, Jinan, China; 14 Department of Hematology/Oncology, KunMing Children’s Hospital, Kunming, China; 15 Department of Hematology/Oncology, Jiangxi Provincial Children’s Hospital, Nanchang, China; 16 Department of Hematology/Oncology, Xi ‘an Northwest Women’s and Children’s Hospital, Xi ‘an, China; 17 Department of Hematology/Oncology, Chongqing Medical University Affiliated Children’s Hospital, Chongqing, China; 18 Department of Pediatrics, Affiliated Hospital of Qingdao University, Qingdao, China; 19 Department of Pediatrics, State Key Laboratory of Experimental Hematology, National Clinical Research Center for Blood Diseases, Institute of Hematology & Blood Diseases Hospital, Chinese Academy of Medical Sciences & Peking Union Medical College, Tianjin, China; 20 Department of Hematology/Oncology, Children’s Hospital of Soochow University, Suzhou, China; 21 Department of Hematology/Oncology, Guangzhou Women and Children’s Medical Center, Guangzhou, China; 22 Department of Pediatrics, Union Hospital of Tongji Medical College, Huazhong University of Science and Technology, Wuhan, China; 23 Departments of Pharmaceutical Sciences, St. Jude Children’s Research Hospital, Memphis, TN USA; 24 Departments of Oncology, Pathology, and Global Pediatric Medicine, St. Jude Children’s Research Hospital, Memphis, TN USA

**Keywords:** Acute lymphocytic leukaemia, Prognosis

## Abstract

The outcomes of children with acute lymphoblastic leukemia (ALL) have been incrementally improved with risk-directed chemotherapy but therapy responses remain heterogeneous. Parameters with added prognostic values are warranted to refine the current risk stratification system and inform appropriate therapies. CD9, implicated by our prior single-center study, holds promise as one such parameter. To determine its precise prognostic significance, we analyzed a nationwide, multicenter, uniformly treated cohort of childhood ALL cases, where CD9 status was defined by flow cytometry on diagnostic samples of 3781 subjects. CD9 was expressed in 88.5% of B-ALL and 27.9% of T-ALL cases. It conferred a lower 5-year EFS and a higher CIR in B-ALL but not in T-ALL patients. The prognostic impact of CD9 was most pronounced in the intermediate/high-risk arms and those with minimal residual diseases, particularly at day 19 of remission induction. The adverse impact of CD9 was confined to specific cytogenetics, notably *BCR::ABL1*^+^ rather than *KMT2A*-rearranged leukemia. Multivariate analyses confirmed CD9 as an independent predictor of both events and relapse. The measurement of CD9 offers insights into patients necessitating intervention, warranting its seamless integration into the diagnostic marker panel to inform risk level and timely introduction of therapeutic intervention for childhood ALL.

## Introduction

Acute lymphoblastic leukemia (ALL) is the most common cancer in children, with an overall cure rate exceeds 90% in most developed countries [1–3]. This remarkable improvement in outcomes can be attributed to the introduction of risk-directed therapies, wherein high-intensity treatments are offered to high-risk patients, and vice versa [4]. Risk stratification in contemporary protocols is based on established prognostic factors such as age, white blood cell count, cytogenetic abnormalities, central nervous system (CNS) involvement, and minimal residual disease (MRD) status [5]. However, treatment responses still vary among patients within the same risk groups [6–8]. While discovery of new genetic subtypes may refine the current risk classification system [9–11], it remains critical to identify additional prognostic markers that can be readily applied in real-world clinical settings.

CD9, a cell surface protein belonging to the tetraspanin superfamily, has been implicated in cancer progression, with its impact on disease outcomes contingent upon the context [12–14]. In B-ALL, previous studies have shown a heterogeneous and subtype-specific expression pattern of CD9 [15, 16] while subsequent investigations showed its enrichment in leukemia-initiating cells [17–19] and involvement in leukemia dissemination [20]. In a recent study involving 153 childhood B-ALL patients treated at a single-center, we reported that CD9 positivity was associated with inferior survival and, when combined with established risk factors such as prednisone response and cytogenetic status, could identify patients at high risk of relapse [21]. However, the study was limited by its small cohort size and treatment heterogeneity, hindered in-depth analyses. To address these limitations, we conducted a retrospective analysis of 3781 subjects treated uniformly under the Chinese Children Cancer Group (CCCG)-ALL-2015 multicenter trial [22]. This comprehensive analysis not only validated previous findings but also unveiled new insights, establishing CD9 as a potential marker for informing prognosis and management of childhood ALL.

## Methods

### Study design and participants

This retrospective cohort study included pediatric patients diagnosed with ALL under the age of 18. Participants were consecutively enrolled in the CCCG-ALL-2015 trial between January 2015 and December 2019. A total of 7640 children from 20 tertiary hospitals in China were recruited for the study. The treatment approach employed in the trial was risk-stratified and guided by MRD assessment. Detailed information regarding the inclusion/exclusion criteria, diagnostic procedures, risk assignment, treatment protocols, and disease monitoring can be found in a previous publication [22]. The institutional ethical committees of all participating centers approved the trial, and informed consent or assent was obtained from the parents, guardians, or patients, as appropriate. This study adhered to the Strengthening the Reporting of Observational Studies in Epidemiology (STROBE) reporting guidelines [23].

### Characterization of CD9 expression

Cell surface CD9 expression was assessed as a lymphoblast marker at the time of diagnosis using flow cytometry. Per study protocol, it is not a mandatory diagnostic marker for ALL. The inclusion of CD9 in the immunophenotyping panel is therefore optional and is an institutional decision based on their laboratory preference as well as marker prioritization depending on sample cellularity. Sixteen sites participated in the measurement of CD9, employing the M-L13 antibody clone conjugated with PerCP-Cy5.5 (12 sites), PE (2 sites), FITC (1 site), or APC-H7 (1 site) obtained from BD Biosciences, San Jose, CA, USA (catalog numbers: 341639, 341637, 341636, 655433). Isotype controls were universally utilized to identify negative cell populations. CD9 positivity was defined as the presence of ≥20% CD9^+^ blasts, following the previously described criteria [21]. To ensure the optimal prognostic performance, various thresholds for CD9 positivity were evaluated through a univariate analysis within this study cohort. The analysis showed that it was prognostically significant for B-ALL at all the cut-offs applied. The selected cut-off (i.e., 20%), which confirmed to have the highest odds and the lowest *P* values for adverse events or relapse, was used for downstream analyses (Supplementary Table 1).

### Outcomes and measures

The aim of this study was to assess the prognostic significance of CD9 and its added value beyond clinical and other biological features in predicting outcomes in childhood ALL within the context of risk-directed therapy. The primary endpoints included 5-year event-free survival (EFS) and cumulative incidence of relapse (CIR) in patients with CD9^+^ or CD9^–^ phenotypes. The secondary endpoints focused on evaluating its precise prognostic features in the context of known risk factors. The outcome data presented in this report were updated as of June 30, 2022. The median follow-up period for the 7042 patients who were alive at the time of analysis was 53.9 months (interquartile range: 40.5–69; range, 0.2–91.8). Among them, 2826 patients were followed for 5 years or more.

### Statistical analysis

EFS was calculated from the date of diagnosis until the occurrence of the initial major event, which included relapse, refractory disease, death from any cause, second malignancy, or off-protocol due to severe toxicity. In the absence of such events, time was treated as censored at the date of last follow-up. Patients who abandoned treatment without severe toxicity, were lost to follow-up, or transferred to other hospitals were censored at the date of their last contact. The 5-year EFS rates were estimated using the Kaplan–Meier method and compared with log-rank test. CIR accounting for competing events was constructed by the method of Kalbfleisch and Prentice [24] and compared with Gray’s test [25]. Categorical variables were compared using Pearson’s *χ*^2^ test or Fisher’s exact test, while continuous variables were compared using Mann–Whitney *U* test. The Cox proportional hazards model was utilized for univariate and multivariate analyses to estimate the hazard ratio and significance of each prognostic factor [26]. Statistical analyses were conducted with R software version 4.3.1 (The R Foundation for Statistical Computing, Vienna, Austria), or SPSS version 25.0 (SPSS Inc., Chicago, IL, USA).

## Results

### Study population

Among 7640 patients enrolled in the clinical trial, CD9 data were available for 3781 patients, accounting for 49.5% of the total cohort. For the 16 centers that participated in CD9 measurement, the data availability rates ranged from 2.9% to 99.6% of recruited subjects. Comparison between the groups with available CD9 data and those without (Supplementary Table 2) revealed no significant differences in age, sex and initial white cell count. However, the CD9 available group showed a higher prevalence of T-ALL, while the distribution of major B-lineage cytogenetic anomalies was similar between the two groups, except for hyperdiploidy (which had a higher prevalence in the CD9 available group) and B-others (which had a lower prevalence in the CD9 available group). Additionally, the CD9 available group exhibited a lower frequency of CNS2 diseases and better MRD responses, leading to a higher proportion of patients being categorized as low risk and fewer patients as high risk.

### Lineage-specific impact of CD9 on childhood ALL

**Fig. 1:**
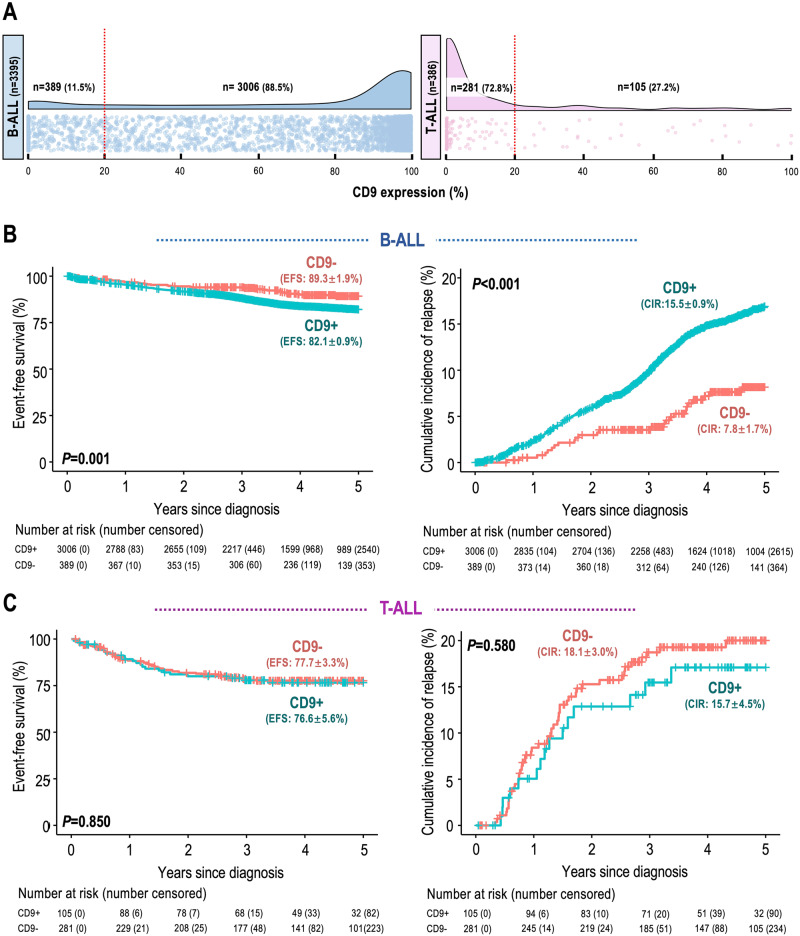
Expression pattern and overall prognostic impact of CD9 in childhood ALL. **A** Raincloud plots showing the distribution of CD9 expression among B-ALL (*n* = 3395) and T-ALL (*n* = 386) cases. The positivity cut-off was set to 20% as determined by univariate analyses. **B**, **C** Kaplan–Meier analyses of 5-year event-free survival (EFS, left panel) and cumulative incidence of relapse (CIR, right panel) in B-ALL and T-ALL patients stratified by CD9 expression status. The number of patients at risk/censored over time, rates of EFS and CIR with standard errors, and *P* values are indicated.

The comprehensive prognostic impact of CD9 was evaluated in the final study cohort of 3395 B-ALL and 386 T-ALL patients. By employing a 20% positivity threshold, 3006 B-ALL (88.5%) and 105 T-ALL (27.2%) cases were classified as CD9^+^ (Fig. 1A). Within the B-lineage cohort, a comparison between CD9^+^ and CD9^–^ patients revealed significantly inferior 5-year EFS for CD9^+^ patients (82.1% vs. 89.3%, *P* = 0.001) (Fig. 1B). Additionally, the CIR was significantly higher in the CD9^+^ group (15.5% vs. 7.8%, *P* < 0.001). Notably, no disparities in the site and time of relapse were observed based on CD9 status (Supplementary Table 3). In contrast, for the T-lineage cohort, there were no discernible differences in EFS or CIR between CD9^+^ and CD9^–^ patients (Fig. 1C) regardless of the positivity cut-offs utilized (Supplementary Table 1).

### Precise prognostic insights of CD9 in B-ALL

We conducted a comprehensive analysis to ascertain the precise prognostic implications of CD9 in B-ALL, initially examining its correlation with clinical and biological variables in 3,006 CD9^+^ and 389 CD9^–^ patients (Table 1). Notably, the CD9^+^ group had a higher proportion of patients with initial white cell count >50 × 10^9^/L (17.9% vs. 8.0%, *P* < 0.001). Hyperdiploidy (18.2% vs. 3.1%, *P* < 0.001) and *TCF3::PBX1* (6.2% vs. 1.0%, *P* < 0.001) were more prevalent in CD9^+^ patients, while *ETV6::RUNX1* was more frequent in CD9^–^ patients (47.3% vs. 18.2%, *P* < 0.001), reinforcing its subtype-specific expression pattern. No significant difference was observed in the CNS disease status and MRD response between CD9^+^ and CD9^–^ groups. Based on these parameters, a higher proportion of CD9^+^ patients were classified as intermediate risk (40.2% vs. 32.1%, *P* = 0.002), while fewer were categorized as low risk (58.7% vs. 66.6%, *P* = 0.003).

**Table 1:** Clinical characteristics of B-ALL patients by CD9 expression.

Clinical parameters	Patients with CD9 data (*n* = 3395)		CD9^+^ patients (*n* = 3006)		CD9^–^ patients (*n* = 389)		CD9^+^ vs. CD9^–^
	*N*	%	*N*	%	*N*	%	*P*
Age, years							
Median	4.3		4.2		4.6		***0.013***^a^
(IQR)	(2.9–6.8)		(2.9–6.7)		(3.2–7.4)		
<1	81	2.4	74	2.5	7	1.8	0.421^b^
1–9.9	2947	86.8	2612	86.9	335	86.1	0.671^b^
≥10	367	10.8	320	10.6	47	12.1	0.390^b^
Sex							
Male	1953	57.5	1721	57.3	232	59.6	0.370^b^
Female	1442	42.5	1285	42.7	157	40.4	
WBC, ×10^9^/L							
Median	8.3		8.7		6.9		***<0.001***^a^
(IQR)	(4.1–28.2)		(4.1–30.0)		(3.9–17.1)		
<50	2825	83.2	2467	82.1	358	92.0	***<0.001***^b^
≥50	570	16.8	539	17.9	31	8.0	
Cytogenetics							
Normal karyotype	981	28.9	878	29.2	103	26.5	0.264^b^
Hyperdiploidy	558	16.4	546	18.2	12	3.1	***<0.001***^c^
* BCR::ABL1*	144	4.2	130	4.3	14	3.6	0.504^b^
* ETV6::RUNX1*	730	21.5	546	18.2	184	47.3	***<0.001***^c^
* KMT2A*-rearranged	129	3.8	116	3.9	13	3.3	0.616^b^
* TCF3::PBX1*	191	5.6	187	6.2	4	1.0	***<0.001***^c^
Others	662	19.5	603	20.1	59	15.2	0.022^b^
CNS status							
CNS1	3162	93.1	2796	93.0	366	94.1	0.431^b^
CNS2	26	0.8	24	0.8	2	0.5	0.761^d^
CNS3	21	0.6	20	0.7	1	0.3	0.502^d^
Traumatic	186	5.5	166	5.5	20	5.1	0.756^b^
Risk group							
Low	2023	59.6	1764	58.7	259	66.6	***0.003***^c^
Intermediate	1332	39.2	1207	40.2	125	32.1	***0.002***^c^
High	40	1.2	35	1.2	5	1.3	0.835^b^
Short-term outcomes							
^ e^D19 MRD^+^	429	13.2	377	13.1	52	13.9	0.646^b^
^ f^D46 MRD^+^	312	10.0	277	10.0	35	9.4	0.943^b^
CR	3346	98.6	2963	98.6	383	98.5	0.862^b^
Induction death	27	0.8	24	0.8	3	0.8	1.000^d^

*IQR* interquartile range, *WBC* white blood cell, *CNS* central nervous system, *MRD* minimal residual disease, *CR* complete remission.

Statistics: ^a^Mann–Whitney *U* test; ^b^Pearson’s *χ*^2^ test; ^c^Pearson’s *χ*^2^ test with Bonferroni correction; ^d^Fisher’s exact test. MRD cut-offs: ^e^D19+, ≥1%; ^f^D46+, ≥0.01%. Missing data: ^e^D19 MRD status (*n* = 141); ^f^D46 MRD status (*n* = 273).

The bold and italic *P* values denote comparisons reaching statistical significance.

We further integrated clinical and biological parameters into the outcome analysis to curate the prognostic impact of CD9 on specific patient subgroups (Table 2). The adverse influence of CD9 on B-ALL was evident in males, patients with low initial white cell count, and those in the intermediate or high-risk group, regardless of age, except for infants. We also scrutinized the prognostic ramifications of CD9 concerning common cytogenetic anomalies. A distinct difference in outcomes was observed for patients with *BCR::ABL1* when stratified by CD9 status, with the absence of CD9 associated with excellent EFS (100% vs. 56%, *P* = 0.019) and CIR (0% vs. 39.5%, *P* = 0.013). Similar favorable outcomes were observed for CD9^–^ patients with normal karyotype (CIR: 6.8% vs. 15%, *P* = 0.034). While not reaching statistical significance, none of the CD9^–^ patients with hyperdiploidy or *TCF3::PBX1* relapsed. However, this pattern was not observed for patients with *ETV6::RUNX1*, *KMT2A*-rearrangements, or not otherwise specified B-ALL. We further integrated MRD status to illuminate the prognostic significance of CD9. In CD9^+^ patients, EFS was inferior in both high (65.2% vs. 82.0%, *P* = 0.053) and low (77.8% vs. 86.6%, *P* = 0.031) day 19 MRD categories. Correspondingly, the CIR was notably elevated in CD9^+^ patients with high (30.3% vs. 9.7%, *P* = 0.007) or low (20.1% vs. 10.8%, *P* = 0.022) MRD on day 19. However, in the analysis based on day 46 MRD status, inferior outcomes were only observed for CD9^+^ patients with negative MRD.

**Table 2:** Precise prognostic features of CD9 in B-ALL.

		5-year EFS, % (95% CI)			5-year CIR, % (95% CI)		
Clinical parameters	*n* (CD9^+/–^)	CD9^+^	CD9^–^	*P*	CD9^+^	CD9^–^	*P*
Age, years							
<1	74/7	64.9 (54.4–77.3)	57.1 (30.1–100)	0.500	24.5 (12.8–34.6)	20.0 (0–48.4)	0.933
1–9.9	2612/335	83.8 (82.0–85.4)	90.5 (87.1–94.0)	***0.004***	14.5 (13.0–16.1)	7.6 (4.3–10.7)	***0.001***
≥10	320/47	71.7 (66.4–77.3)	85.8 (75.7–97.2)	***0.049***	22.1 (16.8–27.1)	8.1 (0–16.6)	***0.027***
Sex							
Male	1721/232	80.3 (78.2–82.5)	89.0 (84.7–93.5)	***0.005***	17.6 (15.5–19.6)	9.4 (5.1–13.4)	***0.004***
Female	1285/157	84.4 (82.3–86.6)	89.7 (84.6–95.1)	0.084	12.9 (10.8–14.9)	5.6 (1.4–9.7)	***0.010***
WBC, ×10^9^/L							
<50	2467/358	84.5 (82.9–86.1)	90.4 (87.1–93.9)	***0.006***	13.5 (12.0–15.1)	7.3 (4.2–10.3)	***0.002***
≥50	539/31	70.6 (66.5–75.0)	76.2 (62.1–93.4)	0.670	25.2 (20.9–29.2)	14.5 (0.3–26.7)	0.238
Risk group							
Low	1764/259	88.3 (86.7–90.0)	92.2 (88.6–95.9)	0.079	10.5 (8.9–12.1)	6.7 (3.2–10.1)	0.061
Intermediate/high	1242/130	72.8 (70.1–75.7)	83.2 (76.5–90.6)	***0.028***	23.1 (20.4–25.8)	10.2 (4.2–15.9)	***0.003***
Cytogenetics							
Normal karyotype	878/103	82.4 (79.7–85.3)	90.4 (84.2–97.2)	0.066	15.0 (12.2–17.6)	6.8 (0.7–12.5)	***0.034***
Hyperdiploidy	546/12	83.9 (80.6–87.4)	100 (100–100)	0.190	14.3 (10.9–17.5)	0 (0–0)	0.189
* BCR::ABL1*	130/14	56.0 (46.0–68.2)	100 (100–100)	***0.019***	39.5 (27.1–49.8)	0 (0–0)	***0.013***
* ETV6::RUNX1*	546/184	90.7 (88.0–93.5)	91.3 (87.0–95.8)	0.953	8.6 (5.9–11.3)	6.4 (2.4–10.2)	0.473
* KMT2A*-rearranged	116/13	61.8 (53.0–72.1)	53.8 (32.6–89.1)	0.416	30.3 (20.6–38.9)	36.4 (0.5–59.3)	0.577
*TCF3::PBX1*	187/4	86.8 (81.9–91.9)	100 (100–100)	0.452	10.7 (6.0–15.1)	0 (0–0)	0.492
Others	603/59	79.1 (75.6–82.8)	84.5 (75.1–95.2)	0.270	18.5 (14.9–21.9)	12.3 (2.4–21.3)	0.188
D19 MRD							
<0.01% (negative)	1537/190	89.1 (87.4–90.8)	93.1 (89.1–97.2)	0.088	9.2 (7.6–10.8)	5.4 (1.6–9.1)	0.070
0.01–0.99% (low)	967/131	77.8 (74.9–80.8)	86.6 (80.5–93.3)	***0.031***	20.1 (17.2–23.0)	10.8 (4.8–16.5)	***0.022***
≥1% (high)	377/52	65.2 (59.9–70.9)	82.0 (71.4–94.3)	0.053	30.3 (24.6–35.6)	9.7 (0.1–18.3)	***0.007***
D46 MRD							
<0.01% (negative)	2491/319	85.5 (83.9–87.0)	92.5 (89.4–95.8)	***0.001***	13.1 (11.6–14.6)	6.5 (3.4–9.5)	***0.001***
0.01–0.99% (low)	246/30	62.0 (55.2–69.6)	71.7 (55.7–92.1)	0.305	36.9 (29.4–43.6)	19.9 (2.5–34.2)	0.079
≥1% (high)	31/5	21.7 (10.3–45.8)	50.0 (18.8–100)	0.287	52.8 (23–71.1)	0 (0–0)	0.300

*EFS* event-free survival, *CIR* cumulative incidence of relapse, *CI* confidence interval.

Statistics: EFS, log-rank test; CIR, Gray’s test.

The bold and italic *P* values denote comparisons reaching statistical significance.

In univariate analysis for treatment outcomes, CD9 positivity emerged as a risk factor for events (HR = 1.748, *P* = 0.001) and relapse (HR = 2.127, *P* < 0.001), alongside other established parameters including age, sex, white cell count, cytogenetic subtypes, CNS status, risk group and MRD response (Supplementary Table 4). Multivariate analysis further confirmed CD9 positivity as an independent risk factor for events (HR = 1.917, *P* = 0.001) and relapse (HR = 2.208, *P* < 0.001) (Table 3).

**Table 3:** Multivariate analysis of treatment outcomes in B-ALL.

	No. of patients (yes/no)	No. of events (yes/no)	HR	95% CI	*P*
Event-free survival					
CD9 positive^a^	3006/389	466/36	1.917	1.313–2.798	***0.001***
Age <1 or ≥10 years	448/2947	114/388	1.548	1.218–1.967	***<0.001***
WBC ≥50 × 10^9^/L	570/2825	145/357	1.559	1.239–1.963	***<0.001***
High-risk group	40/3355	26/476	4.531	2.904–7.070	***<0.001***
* BCR::ABL1* or *KMT2A*r	273/3122	88/414	2.127	1.609–2.813	***<0.001***
CNS2 or CNS3	47/3348	13/489	2.062	1.099–3.871	***0.024***
D19 MRD ≥0.01%	1527/5014	316/634	2.106	1.700–2.609	***<0.001***
D46 MRD ≥0.01%	312/5932	106/774	2.035	1.582–2.618	***<0.001***
Cumulative incidence of relapse					
CD9 positive^a^	3006/389	391/25	2.208	1.435–3.398	***<0.001***
Male gender	1953/1442	268/148	1.289	1.045–1.591	***0.018***
Age <1 or ≥10 years	448/2947	78/338	1.321	1.010–1.729	***0.042***
WBC ≥50 × 10^9^/L	570/2825	117/299	1.561	1.221–1.997	***<0.001***
* BCR::ABL1* or *KMT2A*r	273/3122	70/346	2.190	1.621–2.958	***<0.001***
D19 MRD ≥0.01%	1527/1727	316/159	2.128	1.694–2.673	***<0.001***
D46 MRD ≥0.01%	312/2810	90/296	2.184	1.695–2.814	***<0.001***

Variables for analysis: CD9 positivity, male gender, age (<1 or ≥10 years), diagnostic WBC (≥50 × 10^9^/L), adverse cytogenetics (*BCR::ABL1* fusion or *KMT2A*-rearrangement), risk group (intermediate or high), CNS involvement (CNS2 or CNS3), MRD ≥0.01% at D19 or D46.

Statistics: Cox proportional hazards model with backward stepwise regression.

The bold and italic *P* values denote comparisons reaching statistical significance.

*HR* hazard ratio, *CI* confidence interval.

^a^Remained statistically significant after inclusion of *ETV6::RUNX1* as a covariate.

## Discussion

This study capitalizes on one of the largest clinical trials for childhood ALL reported to date [22] to comprehensively dissect the prognostic implications of CD9. We unveiled CD9’s lineage-specific expression pattern and its survival impact on childhood ALL. This protein was expressed in only 27.2% of T-ALL but significantly in 88.5% of B-ALL cases, likely due to its inherent disparities within the developmental hierarchy of blood lineages [27]. An intriguing observation was that CD9 status affected B-ALL outcome while sparing T-ALL, indicating its context-dependent implications for cancer progression. This could be potentially driven by the hematopoietic lineage-specific difference in the composition of CD9 binding partners within the tetraspanin web, a well-recognized phenomenon in solid cancers to determine its oncogenic *versus* tumor suppressive role that deserves further biological studies in leukemias [12, 13].

In B-ALL, CD9^+^ patients experienced inferior EFS primarily due to increased relapse rates, findings consistently replicated in this extensive patient cohort. The less favorable prognosis of CD9^+^ patients seems linked to the interplay of pre-treatment risk factors. Specifically, CD9^+^ cases had higher WBC at presentation, a recognized unfavorable factor. CD9 was also associated with cytogenetics. For the two most common anomalies, hyperdiploidy occurred mostly in CD9^+^ patients while *ETV6::RUNX1* was predominant in CD9^–^ cases. Notably, when focusing solely on these two genotypes, the CD9^+^ group accounted for only 36.4% patients whereas CD9^–^ patients constituted 50.4%, emphasizing the favorable cytogenetic profile of the latter. Additionally, CD9 positivity was notably linked to intermediate-risk *TCF::PBX1*. This distribution of prognostic parameters already contributed to more CD9^–^ patients falling into the low-risk category. Yet, upon deeper analysis within risk groups, the prognostic potency of CD9 was moderate for low-risk patients but strikingly for the intermediate/high-risk group. Here, CD9^+^ patients faced twice the risk of relapse, underscoring CD9’s potency to pinpoint patients with genuinely high-risk diseases.

With this large cohort of patients, we were able to conduct a comprehensive analysis to discern the distinct impact of CD9 on various cytogenetic subtypes. Notably, among CD9^–^ patients with the *BCR::ABL1* subtype, a remarkable outcome was observed, with a 100% EFS rate. In contrast, CD9^+^ patients had a substantially lower EFS of only 56%. Accordingly, *BCR::ABL1*^+^ patients with a CD9^–^ phenotype could be effectively managed by the current chemotherapy regimen and a tyrosine kinase inhibitor [28]. On the contrary, innovative therapies are warranted for CD9^+^ patients within this subgroup. In future protocols, CD9 positivity with concurrent *BCR::ABL1* may be considered as an indication for allogeneic hematopoietic stem cell transplantation (HSCT) or upfront immunotherapies. Similarly, among patients with a normal karyotype, CD9^–^ patients had a favorable outcome with CIR of only 6.8%, whereas CD9^+^ subjects had a notably higher CIR of 15%, underscoring the necessity for more intensive treatments. While our investigations, alongside those of others, have highlighted CD9’s influence on leukemia stem cell renewal, leukemia-stroma interaction, and leukemia dissemination [17–21], potentially explaining its oncogenic role in B-ALL in general, the differential impact of CD9 on cytogenetic subtypes identified by this study has provided new insights into its underlying biology. It is conceivable that cooperative pathways might be requisite for CD9 to fully manifest its function. For example, our prior work showed that CD9 activates the PI3K/Akt pathway to drive leukemia progression and chemoresistance in *BCR::ABL1*^+^ cells [29]. A systematic dissection of its underlying mechanisms in future studies, possibly by transcriptomic analyses comparing CD9^+^ and CD9^–^ cases in each cytogenetic background, holds the potential for revealing important findings with therapeutic implications.

Importantly, when combined with MRD status, CD9 emerges as a potent prognostic determinant. Notably, our previous single-center study, conducted prior to the introduction of MRD monitoring, failed to capture this informative synergy. Specifically, on day 19 after initiation of induction therapy, CD9^+^ patients who had low or high MRD had a 1.9- and 3.1-fold augmented risk of relapse, culminating in EFS rates of 77.8% and 65.2%, respectively. Consequently, these subjects should be managed early with treatment intensification or innovative therapies to reduce the risk of relapse. However, by day 46, CD9 did not impart an additional value to predict relapse for patients who had already tested positive for MRD. Thus, CD9 should be embraced as an early marker, complementing the specific prognostic parameters identified in this study to inform proper patient management, where HSCT or immunotherapies could be timely introduced for patients with high risk of relapse. Although CD9 appeared to be highly associated with known risk factors, multivariate analyses underscore its autonomy as an independent predictive factor for adverse events and relapse.

This is the largest study to evaluate the significance of CD9 in childhood ALL. A prominent strength is the multicenter design, where all patients underwent treatment following a standardized protocol. This approach generated a wealth of reliable and unbiased data, solidifying the genuine prognostic impact of CD9. Leveraging this substantial cohort, we were able to perform subgroup analyses with other well-established risk factors, meticulously documenting its precise prognostic attributes. However, this study has some limitations. One notable limitation is the absence of standardized flow cytometry protocols across participating centers, specifically in regard to the fluorochromes employed for CD9 detection. Addressing this concern in future trials, guided by Euroflow’s recommendations [30], is essential to enhance consistency and vigor. Another shortcoming pertains to our cohort composition, therein approximately 50% of patients exhibited either a normal karyotype or an ALL categorization marked as “not otherwise specified.” Given the rapidly expanding molecular taxonomy of ALL in the era of genomic medicine [31], it becomes imperative for upcoming studies to incorporate such molecular information. This inclusion would enable a deeper exploration of genetic correlations with CD9 and its subtype-specific prognostic implications.

In sum, we validated the results of our previous single-center study with the findings from this nationwide multicenter study. CD9 positivity unequivocally correlates with a heightened probability of relapse, particularly among patients with intermediate/high-risk diseases, positive MRD status, or specific cytogenetic backgrounds. Notably, *BCR::ABL1*^+^ patients with a CD9^–^ phenotype had excellent outcomes, potentially obviating the necessity for HSCT. Conversely, patients with MRD^+^ who also exhibit CD9 positivity had poor outcomes, underscoring the urgency of early interventions with innovative treatments to mitigate the risk of relapse. As major study groups have yet to mandate the measurement of CD9 [32, 33], we propose its integration into the diagnostic immunophenotyping panel as a ready-to-use prognostic marker to inform risk stratification and management of childhood ALL.

### Supplementary information

Supplementary Information

## Data Availability

The datasets generated during and/or analyzed during the current study are available from the corresponding authors on reasonable request. The data are not publicly available due to privacy or ethical restrictions.
