# Supplementary material for: Prognostic implications of CD9 in childhood acute lymphoblastic leukemia: insights from a nationwide multicenter study in China
Source: Leukemia. 2023 Nov 25;38(2):250–7. doi: 10.1038/s41375-023-02089-3 (PMC10844073; doi:10.1038/s41375-023-02089-3)
Supplement: Supplementary file 1 — Supplementary Information [file 41375_2023_2089_MOESM1_ESM.docx]

**Supplementary Information for Leung et al**

***“Prognostic Implications of CD9 in Childhood Acute Lymphoblastic Leukemia: Insights from a Nationwide Multicenter Study in China”***

**Supplementary Table 1. Prognostically relevant CD9 positivity cut-offs for childhood ALL**

| **EFS** | **B-ALL (n=3395)** | | |  |  | **T-ALL (n=386)** | | |
| --- | --- | --- | --- | --- | --- | --- | --- | --- |
| Cut-off | **HR** | **95% CI** | ***P*** |  |  | **HR** | **95% CI** | ***P*** |
| 10% | 1.678 | 1.122-2.510 | 0.012 |  |  | 1.079 | 0.693-1.681 | 0.737 |
| 20% | 1.771 | 1.250-2.510 | 0.001 |  |  | 1.047 | 0.646-1.698 | 0.851 |
| 30% | 1.598 | 1.192-2.141 | 0.002 |  |  | 1.141 | 0.682-1.907 | 0.616 |
| 40% | 1.498 | 1.150-1.952 | 0.003 |  |  | 1.160 | 0.662-2.032 | 0.604 |
| 50% | 1.351 | 1.067-1.712 | 0.012 |  |  | 0.997 | 0.514-1.933 | 0.993 |
| 60% | 1.330 | 1.066-1.660 | 0.012 |  |  | 0.605 | 0.245-1.496 | 0.277 |
| 70% | 1.350 | 1.096-1.662 | 0.005 |  |  | 0.768 | 0.281-2.098 | 0.606 |
| 80% | 1.357 | 1.113-1.655 | 0.003 |  |  | 0.664 | 0.163-2.702 | 0.567 |
| 90% | 1.228 | 1.020-1.478 | 0.030 |  |  | 0.958 | 0.133-6.883 | 0.966 |
| **CIR** | **B-ALL (n=3395)** | | |  |  | **T-ALL (n=386)** | | |
| Cut-off | **HR** | **95% CI** | ***P*** |  |  | **HR** | **95% CI** | ***P*** |
| 10% | 2.079 | 1.279-3.380 | 0.003 |  |  | 1.011 | 0.607-1.686 | 0.966 |
| 20% | 2.110 | 1.397-3.188 | 0.000 |  |  | 0.849 | 0.475-1.519 | 0.582 |
| 30% | 1.677 | 1.210-2.325 | 0.002 |  |  | 0.889 | 0.474-1.670 | 0.715 |
| 40% | 1.666 | 1.232-2.252 | 0.001 |  |  | 0.864 | 0.426-1.751 | 0.684 |
| 50% | 1.570 | 1.196-2.062 | 0.001 |  |  | 0.911 | 0.415-2.000 | 0.816 |
| 60% | 1.554 | 1.204-2.006 | 0.001 |  |  | 0.648 | 0.235-1.785 | 0.401 |
| 70% | 1.521 | 1.201-1.924 | 0.000 |  |  | 0.756 | 0.237-2.412 | 0.637 |
| 80% | 1.531 | 1.224-1.916 | 0.000 |  |  | 0.419 | 0.058-3.021 | 0.388 |
| 90% | 1.366 | 1.111-1.679 | 0.003 |  |  | 0.049 | 0.000-1340 | 0.563 |

Abbreviations: EFS, event-free survival; CIR, cumulative incidence of relapse; CI, confidence interval. Statistics: Cox proportional hazards model.

**Supplementary Table 2. Clinical characteristics of ALL patients by availability of CD9 data**

| **Clinical Parameters** | **Entire cohort (n = 7640)** | | **With CD9 data (n = 3781)** | | **Without CD9 data (n = 3859)** | | **With *vs* without** |
| --- | --- | --- | --- | --- | --- | --- | --- |
|  | **No.** | **%** | **No.** | **%** | **No.** | **%** | ***P*** |
| Age, years |  |  |  |  |  |  |  |
| Median | 4.6 | | 4.5 | | 4.7 | | ***0.033***^a^ |
| (IQR) | (3.0-7.5) | | (3.0-7.3) | | (3.0-7.7) | |  |
| Sex |  |  |  |  |  |  |  |
| Male | 4521 | 59.2 | 2234 | 59.1 | 2287 | 59.3 | 0.873^b^ |
| Female | 3119 | 40.8 | 1547 | 40.9 | 1572 | 40.7 |  |
| WBC, ×10^9^/L |  |  |  |  |  |  |  |
| Median | 9.7 | | 9.7 | | 9.7 | | 0.525^a^ |
| (IQR) | (4.2-36.5) | | (4.3-37.6) | | (4.2-35.7) | |  |
| Immunophenotype |  | |  | |  | |  |
| B-lineage | 6916 | 90.5 | 3395 | 89.8 | 3521 | 91.2 | ***0.032***^b^ |
| T-lineage | 724 | 9.5 | 386 | 10.2 | 338 | 8.8 |  |
| Subtypes (B-lineage) |  |  |  |  |  |  |  |
| Normal karyotype | 2017 | 29.2 | 981 | 28.9 | 1036 | 29.4 | 0.629^b^ |
| Hyperdiploidy | 1031 | 14.9 | 558 | 16.4 | 473 | 13.4 | ***<0.001***^c^ |
| *BCR::ABL1* | 329 | 4.8 | 144 | 4.2 | 185 | 5.3 | 0.048^b^ |
| *ETV6::RUNX1* | 1452 | 21.0 | 730 | 21.5 | 722 | 20.5 | 0.309^b^ |
| *KMT2A*-rearranged | 223 | 3.2 | 129 | 3.8 | 94 | 2.7 | 0.008^b^ |
| *TCF3::PBX1* | 384 | 5.6 | 191 | 5.6 | 193 | 5.5 | 0.793^b^ |
| Others | 1480 | 21.4 | 662 | 19.4 | 818 | 23.2 | ***<0.001***^c^ |
| CNS status |  |  |  |  |  |  |  |
| CNS1 | 7072 | 92.6 | 3520 | 93.1 | 3552 | 92.0 | 0.080^b^ |
| CNS2 | 118 | 1.5 | 28 | 0.7 | 90 | 2.3 | ***<0.001***^c^ |
| CNS3 | 76 | 1.0 | 31 | 0.8 | 45 | 1.2 | 0.127^b^ |
| Traumatic | 374 | 4.9 | 202 | 5.3 | 172 | 4.5 | 0.073^b^ |
| Risk group |  |  |  |  |  |  |  |
| Low | 3948 | 51.7 | 2023 | 53.5 | 1925 | 49.9 | ***0.002***^c^ |
| Intermediate | 3543 | 46.4 | 1701 | 45.0 | 1842 | 47.7 | 0.016^b^ |
| High | 149 | 2.0 | 57 | 1.5 | 92 | 2.4 | ***0.006***^c^ |
| Short-term outcomes |  |  |  |  |  |  |  |
| ^f^D19 MRD^+^ | 1370 | 18.6 | 581 | 16.1 | 789 | 20.9 | ***<0.001***^b^ |
| ^g^D46 MRD^+^ | 965 | 13.6 | 368 | 10.6 | 597 | 16.5 | ***<0.001***^b^ |
| CR | 7508 | 98.3 | 3721 | 98.4 | 3787 | 98.1 | 0.350^b^ |
| Induction death | 73 | 1.0 | 32 | 0.8 | 41 | 1.1 | 0.327^b^ |
| ^h^Long-term outcomes |  |  |  |  |  |  |  |
| EFS (%, 95% CI) | 81.4 (80.4-82.4) | | 82.4 (81.0-83.7) | | 80.5 (79.0-81.9) | | 0.068^d^ |
| CIR (%, 95% CI) | 15.6 (13.8-17.5) | | 14.9 (12.4-17.7) | | 16.3 (13.7-19.0) | | 0.154^e^ |

Abbreviations: IQR, interquartile range; WBC, white blood cells; CNS, central nervous system; MRD, minimal residual disease; CR, complete remission; EFS, event-free survival; CIR, cumulative incidence of relapse; CI, confidence interval. Statistics: ^a^Mann-Whitney U test; ^b^Pearson’s Chi-square test; ^c^Pearson’s Chi-square test with parameters that remained significant after ‎Bonferroni correction; ^d^log-rank test; ^e^Gray’s test. MRD cut-offs: ^f^D19^+^, ≥1%; ^g^D46^+^, ≥0.01%. Missing data: ^f^D19 MRD status (n=256); ^g^D46 MRD status (n=559). ^h^No statistical differences in separate analyses of B-ALL or T-ALL.

**Supplementary Table 3. Pattern of relapse in B-ALL patients**

| **Clinical Parameters** | **Patients relapsed (n = 416)** | | **CD9^+^ patients (n = 391)** | | **CD9^-^ patients (n = 25)** | | **CD9^+^ *vs* CD9^-^** |
| --- | --- | --- | --- | --- | --- | --- | --- |
|  | **No.** | **%** | **No.** | **%** | **No.** | **%** | ***P*** |
| Time to relapse |  |  |  |  |  |  |  |
| Early^a^ | 278 | 66.8 | 265 | 67.8 | 13 | 52.0 | 0.104 |
| Late^b^ | 138 | 33.2 | 126 | 32.2 | 12 | 48.0 |  |
| Site of relapse^c^ |  |  |  |  |  |  |  |
| Isolated bone marrow | 293 | 72.5 | 272 | 71.8 | 21 | 84.0 | 0.184 |
| Isolated extramedullary | 84 | 20.8 | 81 | 21.4 | 3 | 12.0 | 0.263 |
| Combined | 27 | 6.7 | 26 | 6.8 | 1 | 4.0 | 1.000 |

Statistics: Pearson’s Chi-square test.

Time to relapse: ^a^early relapse, <36 months from diagnosis; ^b^late relapse, ≥36 months from diagnosis.

Missing data: ^c^site of relapse (n=12).

**Supplementary Table 4. Univariate analysis of treatment outcomes** **in B-ALL**

|  |  | 5y EFS | | |  | 5y CIR | | |
| --- | --- | --- | --- | --- | --- | --- | --- | --- |
|  |  | **HR** | **95% CI** | ***P*** |  | **HR** | **95% CI** | ***P*** |
| CD9 positive |  | 1.748 | 1.245-2.453 | ***0.001*** |  | 2.127 | 1.420-3.188 | ***<0.001*** |
| Age <1y or ≥10y |  | 2.248 | 1.825-2.771 | ***<0.001*** |  | 1.758 | 1.374-2.249 | ***<0.001*** |
| Male |  | 1.207 | 1.008-1.445 | ***0.041*** |  | 1.352 | 1.106-1.652 | ***0.003*** |
| WBC ≥50×10^9^/L |  | 2.309 | 1.903-2.800 | ***<0.001*** |  | 2.226 | 1.797-2.756 | ***<0.001*** |
| Normal karyotype |  | 0.964 | 0.794-1.172 | 0.714 |  | 0.929 | 0.749-1.152 | 0.503 |
| Hyperdiploidy |  | 0.841 | 0.658-1.075 | 0.166 |  | 0.884 | 0.679-1.152 | 0.362 |
| *BCR::ABL1* |  | 2.535 | 1.854-3.466 | ***<0.001*** |  | 2.557 | 1.816-3.599 | ***<0.001*** |
| *ETV6::RUNX1* |  | 0.416 | 0.315-0.550 | ***<0.001*** |  | 0.431 | 0.319-0.582 | ***<0.001*** |
| *KMT2A*-rearranged |  | 3.427 | 2.523-4.656 | ***<0.001*** |  | 3.069 | 2.160-4.359 | ***<0.001*** |
| *TCF3::PBX1* |  | 0.857 | 0.568-1.291 | 0.459 |  | 0.827 | 0.522-1.310 | 0.417 |
| Others |  | 1.325 | 1.078-1.628 | ***0.008*** |  | 1.377 | 1.099-1.724 | ***0.005*** |
| CNS1 |  | 0.903 | 0.649-1.257 | 0.545 |  | 0.995 | 0.682-1.451 | 0.977 |
| CNS2 |  | 2.216 | 1.051-4.674 | ***0.037*** |  | 2.292 | 1.024-5.132 | ***0.044*** |
| CNS3 |  | 2.636 | 1.178-5.897 | ***0.018*** |  | 2.008 | 0.750-5.375 | 0.165 |
| Traumatic |  | 0.852 | 0.570-1.274 | 0.436 |  | 0.774 | 0.489-1.227 | 0.276 |
| Low risk |  | 0.368 | 0.307-0.440 | ***<0.001*** |  | 0.407 | 0.335-0.495 | ***<0.001*** |
| Intermediate risk |  | 2.233 | 1.872-2.664 | ***<0.001*** |  | 2.260 | 1.861-2.743 | ***<0.001*** |
| High risk |  | 12.89 | 8.665-19.17 | ***<0.001*** |  | 4.468 | 2.453-8.137 | ***<0.001*** |
| D19 MRD |  |  |  |  |  |  |  |  |
| <0.01% (negative) |  | 0.409 | 0.338-0.495 | ***<0.001*** |  | 0.391 | 0.317-0.482 | ***<0.001*** |
| 0.01%-0.99% (low) |  | 1.434 | 1.195-1.721 | ***<0.001*** |  | 1.570 | 1.288-1.913 | ***<0.001*** |
| ≥1% (high) |  | 2.519 | 2.046-3.102 | ***<0.001*** |  | 2.344 | 1.862-2.952 | ***<0.001*** |
| D46 MRD |  |  |  |  |  |  |  |  |
| <0.01% (negative) |  | 0.275 | 0.221-0.343 | ***<0.001*** |  | 0.299 | 0.236-0.379 | ***<0.001*** |
| 0.01%-0.99% (low) |  | 2.751 | 2.164-3.498 | ***<0.001*** |  | 3.061 | 2.390-3.920 | ***<0.001*** |
| ≥1% (high) |  | 16.32 | 10.80-24.67 | ***<0.001*** |  | 4.963 | 2.723-9.044 | ***<0.001*** |

Abbreviations: EFS, event-free survival; CIR, cumulative incidence of relapse; HR; hazard ratio; CI, confidence interval. Statistics: Cox proportional hazards model.
